# Here, there and everywhere: emotion and mental state talk in different social contexts predicts empathic helping in toddlers

**DOI:** 10.3389/fpsyg.2014.00361

**Published:** 2014-04-29

**Authors:** Jesse Drummond, Elena F. Paul, Whitney E. Waugh, Stuart I. Hammond, Celia A. Brownell

**Affiliations:** Early Social Development Lab, Department of Psychology, University of PittsburghPittsburgh, PA, USA

**Keywords:** socialization, prosocial behavior, emotion and mental state talk, helping, toddlers

## Abstract

A growing body of literature suggests that parents socialize early-emerging prosocial behavior across varied contexts and in subtle yet powerful ways. We focus on discourse about emotions and mental states as one potential socialization mechanism given its conceptual relevance to prosocial behavior and its known positive relations with emotion understanding and social-cognitive development, as well as parents' frequent use of such discourse beginning in infancy. Specifically, we ask how parents' emotion and mental state talk (EMST) with their toddlers relates to toddlers' helping and how these associations vary by context. Children aged 18- to 30-months (*n* = 38) interacted with a parent during book reading and joint play with toys, two everyday contexts that afford parental discussion of emotions and mental states. Children also participated in instrumental and empathic helping tasks. Results revealed that although parents discuss mental states with their children in both contexts, the nature of their talk differs: during book reading parents labeled emotions and mental states significantly more often than during joint play, especially simple affect words (e.g., happy, sad) and explanations or elaborations of emotions; whereas they used more desire talk and mental state words (e.g., think, know) in joint play. Parents' emotion and mental state discourse related to children's empathic, emotion-based helping behavior; however, it did not relate to instrumental, action-based helping. Moreover, relations between parent talk and empathic helping varied by context: children who helped more quickly had parents who labeled emotion and mental states more often during joint play and who elicited this talk more often during book reading. As EMST both varies between contexts and exhibits context-specific associations with empathic prosocial behavior early in development, we conclude that such discourse may be a key form of socialization in emerging prosociality.

## Here, there and everywhere: emotion and mental state talk in different social contexts predicts empathic helping in toddlers

Young children, even in their early years, exhibit a remarkable capacity to act prosocially toward others. Starting in their second year, infants show concern for and comfort others in distress (Dunn, 1988; Zahn-Waxler et al., 1992), help others complete goal-directed actions (Warneken and Tomasello, 2007; Svetlova et al., 2010), cooperate with others (Rheingold, 1982; Brownell et al., 2006; Warneken et al., 2006), and share information and resources with others (Lizkowski et al., 2008; Brownell et al., 2009, 2013). Infants often perform these actions spontaneously and with great enthusiasm (Rheingold, 1982) and with greater frequency and facility as they approach childhood (see Hay and Cook, 2007; and Drummond et al., in press, for reviews). Although some of these behaviors are simply action- or goal-based, many require the child to read and react to the emotions and mental states of others in distress, such as comforting a sad peer by bringing him a toy. This more advanced form of helping relies on a child's attention to the desires and needs of others, understanding of these abstract subjective states, and motivation to address them. Toddlers begin to exhibit this more advanced form of helping around 18 months (Zahn-Waxler et al., 1979; Dunn, 1988) and are fairly proficient at 30 months (Svetlova et al., 2010). Although the positive slope of this trajectory may be expected, the general time-frame is more puzzling: these behaviors emerge when children's social-cognitive abilities and motivational systems are still immature (Svetlova et al., 2010), raising questions about developmental mechanisms. Theoretical tradition and recent empirical work have established the importance of parental socialization in the development of prosocial functioning, but understanding of the relevant processes and how they operate in the very early development of prosociality remains rudimentary. The purpose of the current paper is to inform this understanding by examining parent discourse about emotions and mental state as one such process.

### Parental socialization of prosocial behavior

Socialization plays a central role in many theories of prosocial development, from modeling of empathic and responsive behavior to direct instruction, guided participation in everyday chores (Rheingold, 1982; Hammond, 2011), and affectively-laden guilt inductions (Hoffman, 2000). These and other processes have been shown to promote prosocial responding in preschool- and school-aged children (see Grusec et al., 2002; and Hastings et al., 2007, for reviews). A small body of literature suggests that parents begin to socialize prosocial behavior in infancy through both global and specific mechanisms. Warm and sensitive responding to a child's needs has been established as a robust contributor to empathic concern and prosocial behavior in 1- and 2-year-olds (Zahn-Waxler and Radke-Yarrow, 1990; Zahn-Waxler et al., 1992; Moreno et al., 2008) and has been shown to predict the trajectory of prosocial behavior into childhood (Robinson et al., 1994). Parents also socialize prosociality by scaffolding their children's participation in everyday household tasks and chores (Rheingold, 1982), which is associated with greater toddler helping and sharing in subsequent prosocial tasks (Hammond, 2011; Pettygrove et al., 2013).

### Prosocial behavior and discourse about emotions and mental states

One potentially important contributor to early prosocial behavior may be parents' discourse about others' emotions and mental states with their young children (henceforth referred to as emotion and mental state talk, EMST). Parents use a wide variety of emotion and mental state vocabulary in conversation with young children, including simple affect (e.g., happy, sad), desire (e.g., want, need), and mental state terms (e.g., think, know) (Beeghly et al., 1986; Dunn et al., 1987; Ensor and Hughes, 2008), and they shape their EMST to match the child's developmental level (Fivush et al., 2006; Taumoepeau and Ruffman, 2006, 2008; Brownell et al., 2013). Developmentally sensitive discourse about emotions and mental states provides children a framework within which to objectify and reflect on abstract subjective concepts, as well as recognize their role in motivating behavior (Bartsch and Wellman, 1995; Taumoepeau and Ruffman, 2006; Ensor and Hughes, 2008). Beginning in late infancy, children use these conversations to gradually construct a more complete understanding of emotions and mental states (Carpendale and Lewis, 2004; Fivush et al., 2006; Taumoepeau and Ruffman, 2006).

A wealth of empirical findings supports the assertion that discourse about emotions and mental states contributes to social and emotion understanding, measured either concurrently or longitudinally, among both preschoolers (Dunn et al., 1991a; Denham et al., 1994; Denham and Auerbach, 1995; Garner et al., 1997; Hughes and Dunn, 1998; Garner, 2003; Ensor and Hughes, 2008) and toddlers (Dunn et al., 1991a,b; Laible and Thompson, 2000; Laible, 2004; Taumoepeau and Ruffman, 2006, 2008; Ensor and Hughes, 2008). A handful of experimental studies have demonstrated the causal link by training parents to use EMST and finding increased emotion understanding or false belief reasoning in their children relative to controls (Guajardo and Watson, 2002; Lohman and Tomasello, 2003; Gavazzi and Ornaghi, 2011; Ornaghi et al., 2011). Thus, it is clear that EMST is a mechanism by which parents socialize normative social understanding beginning in the second year of life.

There is also evidence that the role of EMST extends to prosocial behavior, especially insofar as prosocial responses rely on the ability to attend to, understand, and respond to the emotions and desires of others. Parent-child discourse about emotions and mental states is positively related to prosocial behavior in preschoolers and older children (Denham et al., 1992; Laible and Thompson, 2000; Ruffman et al., 2006; Garner et al., 2008; Ensor et al., 2011) and the research with younger children, albeit limited, suggests similar associations. Zahn-Waxler et al. (1979) reported that children whose mothers accompanied the explanations of their distress with intense feelings, reactions, and disappointments were more likely to show concern toward another in distress and attempt to comfort him. Similarly, Garner (2003) found that toddlers whose parents used more mental state talk when caring for a distressed doll were more likely to subsequently attend to and make sympathetic comments toward an adult whose favorite toy broke. Recently, Brownell et al. (2013) found that parents who used more EMST when reading a wordless picture book with toddlers had children who helped and shared more quickly and more often with an adult in need.

Beyond these general relationships, certain patterns of EMST may play unique roles in prosocial development. Brownell et al. (2013) found that affect terms (e.g., happy, sad) and mental state terms (e.g., think, know) were more strongly related to prosocial behavior than were desire terms (e.g., want, need). Moreover, they found that, over and above the amount of parent talk about emotions and mental states, how much parents *elicited* EMST from children by asking open ended questions about emotions and mental states (e.g., “how does he feel?”), rather than simply labeling and explaining these concepts, predicted subsequent prosocial behavior; actively engaging a child in conversation about emotions appears to provide especially fertile opportunities for the child to attend to these mental states, learn about them, and/or understand how to respond. In fact, parents' use of this elaborative and engaging style may be a crucial process within general parent-child discourse that provides both the required information to the child and a framework within which the child can co-construct social understanding with her parent (for a review, see Fivush et al., 2006).

Finally, EMST may contribute to the development of some aspects of prosocial behavior more than others. Prosocial behavior is a multidimensional construct comprised of many distinct behaviors that rely on different capabilities and stem from different developmental mechanisms (Svetlova et al., 2010; Dunfield et al., 2011; Paulus et al., 2013). Brownell et al. (2013) found that EMST predicted emotion-based helping, which requires an understanding of the recipient's internal state (e.g., bringing a crying friend a toy to cheer her up), but not simple goal-directed helping that does not rely on the same recognition and understanding of affect (e.g., handing someone a marker he has dropped while coloring). These findings suggest that the role of EMST in socializing early helping behavior may be especially relevant for emotion-based prosocial behavior. The primary goal of the current study is to further elucidate the nature of the specific relations between parental EMST and prosocial behavior in infancy when prosocial behavior is first emerging.

### Emotion and mental state talk in context

Parents use discourse about emotions and mental states in many interactive contexts, ranging from pretend play (Dunn et al., 1987; Hughes and Dunn, 1997), conversation about past events (Dunn et al., 1987; Laible and Thompson, 2000; Lagattuta and Wellman, 2002; Laible, 2004), and meal preparation (Ensor and Hughes, 2008) to book reading (Ruffman et al., 2006; Taumoepeau and Ruffman, 2006, 2008; Brownell et al., 2013) and free play (Degotardi and Torr, 2007; Slaughter et al., 2008; Laranjo et al., 2010). Although all offer opportunities to explore emotions and mental states, some situations may better support EMST or more effectively foster the development of social understanding and behavior than others (de Rosnay and Hughes, 2006; Howe et al., 2010). For example, EMST is more frequent and elaborate when parents discuss negative rather than positive emotions (Lagattuta and Wellman, 2002; Fivush et al., 2006), read books (Sabbagh and Callanan, 1998), or eat a snack (Beeghly et al., 1986), and play together with toys rather than without toys (Laranjo et al., 2010). Parents' use of particular subtypes of EMST (e.g., emotion terms vs. mental state terms) also vary by context. For example, Howe et al. (2010) found that parents used more emotion talk with their preschoolers while discussing affectively-charged pictures (similar to those found in picture-books) than during naturally-occurring conversations in the home; but they used more cognitive terms during positively-valenced conversations in the home than while discussing the pictures.

These context differences may be due, in part, to different goals. Parents may use ordinary conversation to help their children learn and adopt socially-appropriate behaviors, while they may be more likely during book-reading to actively try to help their children identify and understand the emotions and internal states depicted in the story (Howe et al., 2010). Additionally, picture books may especially afford emotion talk by introducing an assortment of emotions that are rarely confronted otherwise: the average book for 3–4 year olds contains 17 textual references to mental states, about one reference every three sentences, with nine unique mental states or emotions introduced (Dyer et al., 2000). Particularly evocative illustrations can make abstract mental states more tangible and may be one reason why picture books are helpful in facilitating emotion-related discussions with infants whose limited language capacities might otherwise preclude them from engaging in such conversation (Taumoepeau and Ruffman, 2006, 2008; Slaughter et al., 2007; Brownell et al., 2013).

In addition to context differences in parents' use of EMST, child outcomes may be differentially associated with EMST in particular contexts (Fivush et al., 2006). Discussion of children's emotions in response to past transgressions, for example, may relate more strongly to the development of conscience than discussion of emotions in neutral contexts (Laible and Thompson, 2000, 2002). Moreover, such discussions predicted children's conscience development when the discussions occurred in the lab, but not when they occurred in the home (Laible and Thompson, 2002). Whether context similarly moderates relations between EMST and prosocial development is still unknown. In the current study, we address this gap by comparing parent EMST during book reading and joint play, two ubiquitous parent-child activities during which parents have ample opportunity to discuss emotions and mental states, and examining their unique associations with prosocial behavior. As emotions and mental states are perhaps more tangible and accessible to children when they are visually depicted in a picture book (as noted above), we tentatively hypothesize that conversations about these abstract concepts will be more impactful, and consequently more strongly associated with prosocial behavior, in the context of book-reading as compared to joint play.

### The current study

The current study aims to build on the conceptual and empirical work outlined above by evaluating EMST as a predictor of prosociality during the period when emergent prosocial behaviors are undergoing rapid change. We expect that children whose parents more frequently use EMST, and in particular who elicit EMST from their children, will be more helpful; and we expect findings to be stronger for empathic helping than for instrumental helping. A secondary aim is to compare parental EMST across two distinct interactive contexts, i.e., joint picture-book reading and joint play, and to determine whether relations with prosocial behavior vary by context. We expect parents to use EMST more frequently during book reading than joint play, and we expect EMST in book reading to be more strongly associated with helping than EMST during toy play.

## Methods

### Participants

Forty-four parent-child dyads participated in a larger study of prosocial behavior; 21 18- and 23 30-month-olds. Data from six children were not usable because of procedural error (*n* = 2) or the child's refusal to complete the book-reading task (*n* = 4). The final sample consisted of 38 parent-child dyads; 16 with 18-month-olds (*M* = 18.73; 10 boys and 6 girls) and 22 with 30-month-olds (*M* = 28.87; 13 boys and 9 girls). The sample size, although somewhat small, is consistent with those in other recent studies of early prosocial behavior (e.g., Warneken and Tomasello, 2006; Over and Carpenter, 2009; Brownell et al., 2013). Children were healthy and typically-developing, from working- and middle-class families recruited from a medium-sized mid-Atlantic city and surrounding suburbs. Thirty-five children (92%) were Caucasian; remaining children (one each) were Hispanic, biracial, or unspecified.

### General procedure

Procedures took place in a large playroom. Video was captured from behind a one-way mirror and audio was recorded by an in-room multi-directional microphone hung from the ceiling. The sessions began with a short warm-up to familiarize the child with the lab setting and with the experimenter (E) and an assistant experimenter (AE). Two tasks measuring parental EMST were administered: joint parent-child book reading and parent-child joint play with a standard set of age-appropriate toys. Two helping tasks were administered, one instrumental or action-based, and one empathic or emotion-based, adapted from Svetlova et al. (2010). The parent-child book-reading task was always administered between the two helping tasks (to maximize child participation), which were counterbalanced for order, and the free-play task was always administered last. Parents signed informed consent forms prior to the start of the session. They remained with their children at all times and completed questionnaires while the children engaged in the helping tasks with E. Questionnaires included standard demographic information and the MacArthur Communicative Development Inventory, a well-validated and widely-used measure of early language development (Fenson et al., 2000), which was used as a covariate. All procedures conformed to SRCD ethical standards for research with children and were approved by the university's Institutional Review Board.

### Parent emotion and mental state talk tasks

#### Book reading

E gave a book to the parent, encouraged the parent to read the book as she normally would at home, and left the room. The book was read by the child's regular daytime caregiver who accompanied them to the lab, most often mothers (*n* = 35) but occasionally fathers (*n* = 3). Supplemental analyses showed no differences as a function of who read the books. The book used in this task (Alborough, 2000) included rich emotional content as well as multiple scenes and objects that parents could talk about in addition to or instead of emotions; furthermore, the paucity of words in the book (only three words appear) encouraged parents to speak naturally and without external influence. The content of the book therefore permitted the expression of individual differences in parents' predilection to discuss emotions with their children.

#### Joint play

The parent and child were presented with a basket of age-appropriate toys and given 7 min to play as they typically would at home. E did not give the parent any specific instructions on how to play with the child; this context thus approximates the many unstructured everyday interactions parents have with their children and provides a complement to the more structured interactions captured in the book-reading context.

#### Coding

Parents' language during book reading and joint play was transcribed verbatim from the video records and separated into distinct utterances. As defined by Slaughter et al. (2007), an utterance was considered an uninterrupted stream of language, and utterances were distinguished based on lengthy pauses, grammatical structure, and changes in vocal intonation. Talk unrelated to the book or play, such as correcting a misbehaving child, was not included in transcriptions. Transcription reliability was established on 34% of records; percent agreement was 80%.

Transcripts were coded for six different *content* categories of EMST based on previous research (Ruffman et al., 2006; Symons et al., 2006; Brownell et al., 2013): simple affect talk (e.g., happy, sad, angry), desire talk (e.g., he wants), emotion explanations/elaborations (e.g., he's sad *because he is alone*), other internal state talk (e.g., sick, tired, hungry), mental state talk (e.g., think, know, remember), and empathy statements (e.g., poor guy) (see Supplementary Materials for details).

An additional distinction was made based on the *function* of the talk. Parents' *production* of EMST (labeling or explaining; e.g., “the monkey is sad”) was distinguished from parents' *elicitation* of EMST (asking the child to label or explain' e.g., “how does the monkey feel?”). These different forms of EMST serve different functions (primarily to communicate or elicit information, respectively), make different demands on children's understanding, and may differentially predict outcomes (Ninio, 1980, 1983; Martin and Green, 2005; Brownell et al., 2013).

Thus, each transcript was coded for: total number of utterances; simple affect talk (produced vs. elicited); desire talk (produced vs. elicited); emotion explanations/elaborations (produced vs. elicited); other internal state talk (produced vs. elicited), mental state talk (produced vs. elicited), and empathy statements (produced; no elicitations occurred). Interrater reliability between the first and second authors was established using Cohen's kappa and was excellent for both book reading (κ = 0.92; calculated on 21% of records) and joint play (κ = 0.95; 18% of records). Disagreements were resolved by consensus.

Several composite variables were created to serve as the measures for analysis. Total frequency of EMST utterances was summed within each context, yielding *total EMST book-reading* and *total EMST joint-play*. Total production (sum of simple affect, desire, emotion explanation/elaboration, other internal state, mental state, and empathy statement productions) and total elicitation (sum of simple affect talk elicitation, etc.) were calculated separately, yielding four composite variables: *EMST production book-reading*, *EMST production joint-play*, *EMST elicitation book-reading*, and *EMST elicitation joint-play*. Content categories of EMST (simple affect, desire, emotion explanations/elaborations, other internal states, mental states, and empathy statements) were also summed within each context, yielding composite variables for *simple affect talk: book-reading*, *simple affect talk: joint-play*, *desire talk: book-reading*, and so on. These measures were converted to proportions of total utterances to control for the slightly different amounts of time spent reading the book and playing with the toys. Additionally, the number of different content categories used by each parent was calculated, yielding a score for *number of different content categories*, ranging from 0–6 in each context. Finally, the total number of utterances for each parent in each context was converted to a per-minute rate to account for slight differences in total time spent reading and playing; this *total utterance* rate was used to capture parents' general talkativeness.

### Helping tasks

Children engaged in two tasks with E designed to measure different types of helping behavior: an instrumental helping task and an empathic helping task. These tasks have been used effectively to measure helping behavior in children between 18 and 30 months of age (Warneken and Tomasello, 2006; Over and Carpenter, 2009; Svetlova et al., 2010). The instrumental helping task was designed to measure children's helping behavior with respect to goal-directed actions (picking up sticks accidentally dropped on the floor) and did not require a complex understanding of the recipient's emotional mental state. The empathic helping task required the child to read and understand E's internal state in order to comprehend his need and assist him in alleviating his distress (bringing E a blanket when he shivered with cold, which E had previously modeled by wrapping in a blanket after suddenly shivering). In both tasks, E experienced a distressing event (dropping sticks or becoming cold). After each event, E delivered four cues about his need that communicated progressively more information about what the distress was and how the child could alleviate it. The first cue (E says “oops” or begins to shiver) conveyed the distress. The next cue (E says “I dropped my stick” or “I'm cold”) included a more explicit description of the nature of the distress. The third cue (E says “I dropped my sticks, I need them back/I'm cold, I need my blanket” and reaches twice palm-down for the target object) provided a more explicit description of the need and a way to alleviate it. The fourth and final cue (E reaches palm-up for the target object and asks the child “[child's name], can you help me get my sticks/blanket?”) was the most direct communication about how to help. The child was given 10 s after each cue to help. Cues were stopped after a child helped.

Helping was scored when the child gave the target object to E. Children received a helping score of 0–5 for each task according to the cue at which they helped (0 = did not help; 1 = helped at the last cue; 5 = helped immediately upon E's first cue). High scores thus indicated earlier, more skilled helping, with fewer cues. Percent agreement was calculated between each coder and the primary coder on 20% of the video records, with 100% agreement.

## Results

The primary goal of the current study was to examine associations between parents' EMST and children's instrumental and empathic helping, considered as a function of interactive context. We present the results first for context and age effects on parental EMST, followed by analyses for associations between helping and EMST within each context.

### Context and age differences in emotion and mental state talk

To examine context and age differences in the *function* and *content* of EMST, a series of three-way repeated-measures ANOVAs was conducted on the measures of EMST with context (book-reading; free-play) as the within-subjects factor, and age (18; 30 months) and gender as between-subjects factors (see Table 1 for descriptive statistics and significance tests). Analyses revealed a few unsystematic two- or three-way interactions among gender, age, and context (eight significant interactions out of 68 possible) and will not be reported here (results available from corresponding author). In particular, there were no systematic interactions with age.

**Table 1:** **Descriptive statistics for EMST Proportions as a function of gender, age, and context**.

	**Gender differences**			**Age differences**			**Context differences**		
	**Males (*n* = 23)**	**Females (*n* = 15)**	***F***	**18-mos (*n* = 16)**	**30-mos (*n* = 22)**	***F***	**Book reading**	**Free-play**	***F***
Total utterances (rate per minute)	15.21 (4.27)	17.15 (3.81)	1.98	15.08 (4.43)	16.62 (3.91)	1.77	19.36 (5.20)	14.58 (4.45)	35.26^***^
Total EMST (proportion)	0.16 (0.07)	0.21 (0.08)	4.11^†^	0.15 (0.07)	0.19 (0.08)	1.67	0.18 (0.11)	0.17 (0.08)	1.41
**FUNCTION (PROPORTIONS)**									
EMST elicitations	0.09 (0.06)	0.13 (0.05)	4.83^*^	0.09 (0.05)	0.11 (0.06)	0.41	0.09 (0.08)	0.11 (0.07)	0.73
EMST productions	0.07 (0.04)	0.08 (0.05)	0.50	0.06 (0.03)	0.09 (0.05)	1.68	0.09 (0.07)	0.06 (0.04)	10.98^*^
**CONTENT (PROPORTIONS)**									
Simple affect	0.03 (0.03)	0.04 (0.02)	2.57	0.03 (0.02)	0.05 (0.03)	2.83	0.07 (0.05)	0.01 (0.02)	55.37^***^
Desire talk	0.05 (0.03)	0.06 (0.04)	2.64	0.06 (0.04)	0.05 (0.03)	0.40	0.03 (0.03)	0.07 (0.05)	20.81^***^
Explanations	0.01 (0.01)	0.02 (0.02)	3.94^†^	0.01 (0.01)	0.01 (0.01)	0.61	0.02 (0.03)	0.00 (0.00)	21.21^***^
Mental states	0.03 (0.03)	0.06 (0.03)	5.84^*^	0.03 (0.03)	0.05 (0.03)	9.81^**^	0.03 (0.04)	0.05 (0.04)	4.94^*^
Other internal states	0.04 (0.03)	0.02 (0.02)	1.16	0.03 (0.03)	0.03 (0.03)	0.22	0.03 (0.03)	0.03 (0.03)	0.57
Empathy statements	0.00 (0.01)	0.00 (0.01)	0.01	0.00 (0.01)	0.00 (0.01)	0.04	0.01 (0.01)	0.00 (0.01)	1.73
Number of different content categories	3.04 (1.23)	3.60 (1.06)	1.52	3.06 (1.15)	3.41 (1.21)	1.01	3.55 (1.75)	2.97 (1.05)	6.697^*^

† p = < 0.1;

* p < 0.05;

** p < 0.01;

*** *p < 0.001*.

*Proportions were calculated per total number of parental utterances*.

There were no significant age differences for the rate of total utterances or the proportions of total EMST, EMST elicitations, or EMST productions (see Table 1), indicating that parents discussed emotions and mental states at similar rates with 18- and 30-month old children. However, there were main effects of age in the *content* of mental state talk (simple affect, desires, etc.): parents of 30-month-old children used a significantly higher proportion of mental state terms (e.g., think, know) than did parents of 18-month-olds. No gender differences emerged for the overall rate of parental utterances, but parents used a higher proportion of EMST when talking to girls than when talking to boys. Regarding *function* (productions; elicitations), parents of girls elicited EMST proportionally more than did parents of boys. Regarding *content*, parents of girls used a significantly higher proportion of mental state terms and a marginally higher proportion of emotion explanations/elaborations (e.g., he is sad *because he is alone*) than did parents of boys.

There were several significant context differences (see Table 1). Parents generated utterances at a significantly higher rate during book reading than during joint play, but the proportion of EMST did not differ between contexts. Regarding *function*, parents produced a significantly higher proportion of EMST in the book-reading context than in the free-play context, but there was no significant context difference for EMST elicitations. Regarding the *content* of mental state talk, parents used significantly higher proportions of simple affect talk (e.g., happy/sad) and emotion explanations/elaborations in the book-reading than the free-play context. In contrast, parents used significantly higher proportions of desire talk (e.g., want/need) and mental state talk during joint play than during book reading. Parents also used more distinct content categories in book reading than in joint play.

To examine consistency of EMST across contexts, partial correlations, controlling for age and gender, were conducted to examine associations between EMST productions, EMST elicitations, and total EMST across the two contexts. Analyses revealed significant relations for production of EMST across contexts (partial *r* = 0.33, *p* < 0.05), but not elicitation of EMST (partial *r* = 0.02, *ns*) or total EMST (partial *r* = 0.21, *ns*). There were no significant correlations across contexts for the *content* of EMST. However, the number of different content categories used was significantly correlated across contexts (partial *r* = 0.40, *p* < 0.05).

In sum, parents talked more while reading books with their toddlers than while playing together with toys, although they used the same proportion of EMST in both contexts. They produced proportionally more emotion and mental state labels and explanations during book reading than during joint play, but they asked their children to discuss mental states and emotions to the same degree in both contexts. Parents talked about more distinct EMST *content* categories during book reading than while playing with toys, and those parents who talked about a wider variety of EMST content in one context also did so in the other.

### Helping in relation to discourse about emotions and mental states

To examine associations between parental EMST and children's helping, partial correlations, controlling for age and gender, were calculated between children's instrumental and empathic helping scores and the measures of parental EMST (see Tables 2 and 3). Because age was correlated with children's vocabulary (MacArthur CDI total score; *r* = 0.60, *p* < 0.001), controlling for age also controlled for language differences and other unmeasured characteristics associated with age such as attention, compliance, amount of exposure to books, and so on. Analyses were conducted separately by context to detect any context-specific patterns. Significant correlations were found between empathic helping scores and total EMST during book reading but not joint play. No significant correlations were found between instrumental helping scores and total EMST in either context.

**Table 2:** **Descriptive statistics and significant tests for helping scores as a function of gender, age**.

	**Gender**			**Age**		
	**Males (*n* = 23)**	**Females (*n* = 15)**	***F***	**18-mos (*n* = 16)**	**30-mos (*n* = 22)**	***F***
Instrumental	2.39 (1.64)	2.60 (1.81)	0.14	1.63 (1.67)	3.09 (1.44)	8.38^**^
Empathic	1.35 (1.30)	1.5 (1.35)	0.12	0.53 (1.13)	2.00 (1.07)	16.09^***^

^†^p = < 0.1, ^*^p < 0.05,

** *p < 0.01*,

*** *p < 0.001*.

**Table 3:** **Partial correlations, controlling for age (in months) and gender, between proportions of EMST and helping scores**.

	**Instrumental**	**Empathic**
**BOOK READING**		
Total EMST	−0.26	0.48^**^
**Function**		
EMST elicitations	−0.26	0.36^*^
EMST productions	−0.11	0.31^†^
**Content**		
Simple affect	−0.34^*^	0.26
Desire talk	−0.06	0.08
Emotion explanation	−0.15	0.33^†^
Mental state	−0.12	0.59^***^
Other internal state	0.01	−0.02
Empathy statements	−0.11	0.37^*^
**JOINT PLAY**		
Total EMST	−0.33^†^	0.10
**Function**		
EMST elicitations	−0.25	−0.13
EMST productions	−0.23	0.43^*^
**Content**		
Simple affect	−0.26	0.25
Desire talk	−0.14	−0.16
Emotion explanation	−0.16	−0.05
Mental state	−0.21	0.13
Other internal state	−0.28^†^	0.26
Empathy statements	0.03	0.11

† *p = < 0.1*,

* *p < 0.05*,

** *p < 0.01*,

*** *p < 0.001*.

Regarding *function* of EMST (productions; elicitations): empathic helping scores were correlated significantly with EMST elicitation and marginally with EMST production in the book-reading context (see Table 3). The reverse was true for the joint play context: empathic helping scores were significantly correlated with EMST production, but not with EMST elicitation. No significant correlations were found between instrumental helping scores and elicitation or production of EMST in either context. Regarding *content* of EMST (simple affect, desires, etc.) in the book-reading context, empathic helping scores were significantly positively related to proportion of mental state talk and empathy statements (e.g., poor guy), and marginally with emotion explanations/elaborations; instrumental helping scores were significantly negatively related to simple affect talk (see Table 3). For EMST in the joint play context, empathic helping scores were not significantly related to any content category, while instrumental helping scores were marginally negatively related to proportion of other internal state talk (e.g., hungry).

To determine if eliciting children's talk about emotions during book reading was uniquely associated with children's helping over and above parents' production of EMST (following Brownell et al., 2013), hierarchical linear regression analysis was conducted predicting children's empathic helping scores. As instrumental helping was not significantly related to EMST production or elicitation, no model was run for instrumental helping scores. Age and gender were entered on the first step, followed by parents' EMST production, then parents' EMST elicitation. The full model explained 42% of variance in empathic helping scores, *F*_(4, 32)_ = 5.90, *p* < 0.01, with 27% due to age and gender, *F*_change_(2, 34) = 6.26, *p* < 0.01. EMST production did not account for significant additional variance, but EMST elicitation increased the variance explained by 9%, *F*_change_(1, 32) = 4.81, *p* < 0.05. Thus, parental *eliciting* of children's EMST while reading books together contributed uniquely to toddlers' empathic helping, predicting it above and beyond child age, gender, and parental *production* of EMST.

A parallel regression analysis was conducted on parental EMST in the free-play context. The full model again explained 42% of the variance in empathic helping scores, *F*_(4, 32)_ = 5.83, *p* < 0.01, with 27% due to age and gender, *F*_change_(2, 34) = 6.26, *p* < 0.01. *Production* of EMST increased the variance explained by 14% *F*_change_(1, 33) = 7.94, *p* < 0.01, but *elicitation* of EMST did not add significant variance to empathic helping scores. Thus, parental *production* of EMST during joint play contributed uniquely to toddlers' empathic helping, predicting it above and beyond child age and gender, while parental *elicitation* of EMST from their children during joint play was not predictive.

## Discussion

The goals of the current study were to explore parent-child talk about emotions and mental states in two interactive contexts and to examine such discourse as a potential socialization mechanism in the development of prosocial behavior in 18- and 30-month olds, when prosociality is emerging and undergoing dramatic change. Parent talk was measured while parents and their children read picture books and played with toys, activities chosen for their ecological validity and the opportunities they provide for discussion of emotions and mental states.

Several findings are worth noting. First, parents discussed emotions and mental states with their children at both ages and in both contexts to the same degree, with nearly 20% of their discourse comprised of EMST. Thus, even when their children are very young and likely have a tenuous grasp on abstract mental states, parents devote a significant proportion of their conversation to these concepts. Second, the nature of parents' talk differed in the two contexts. Although they asked children to label or explain emotions and mental states equivalently in both contexts, parents themselves labeled and explained emotions and mental states significantly more while reading a picture book with their children than when playing together with toys. They also varied the content of their EMST more while reading books than playing with toys, discussing more distinct internal states than they did while playing with toys. Conversely, when playing with toys parents used more desire talk (e.g., want, need) and mental state words (e.g., think, know) than they did while reading books with children. Converging with these findings are the abundance of overt affective cues and emotional terms in children's books (Dyer et al., 2000), reflected in our data by a significantly higher proportion of simple affect talk and emotion explanations in the book-reading than the free-play context. These results indicate that toddlers are exposed to a greater overall amount and variability of EMST while reading books with their parents than while engaged in joint toy play. They suggest that although book reading may provide a richer and denser scaffolding experience for the young child, EMST during joint play complements children's exposure to EMST in book reading; this may be especially important for families in which play constitutes a more regular aspect of parent-child interaction than does book reading. Moreover, the nature of parents' talk in one context was generally unrelated to their talk in the other, suggesting that parents tailor their conversational approach to the current interactional setting with toddlers, just as they do with older children (Fivush et al., 2006; Howe et al., 2010).

Importantly, parents' discourse about emotions and mental states in each context was positively related to toddlers' empathic helping. Furthermore, when taken together, EMST production and elicitation during book reading accounted for the same proportion of variance in children's empathic helping scores as when it occurred during joint play. However, associations were more consistent for EMST during book reading, and specific associations varied by context: children who helped more quickly in the empathic helping situation had parents who *elicited* emotion or mental state talk more often from children while they read books together (see also Brownell et al., 2013), but who *labeled* emotions or mental states more often while playing with toys together. Conversely, parents' talk about emotions and mental states was mostly unrelated, and in one case negatively related, to children's goal-directed, instrumental helping. The few unsystematic associations with instrumental helping may result from the ubiquity and relative ease with which toddlers at this age can accomplish goal-based helping tasks (Svetlova et al., 2010; Brownell et al., 2013); alternatively, they may reflect underlying qualitative differences between distinct prosocial behaviors that stem from different mechanisms and rely on different capabilities (Svetlova et al., 2010; Dunfield et al., 2011; Paulus et al., 2013).

This is the first empirical examination, to our knowledge, to evaluate potential context-specific associations between EMST and early-appearing prosocial behavior. The findings add to current understanding by suggesting that both the quantity and quality of EMST play important roles in socializing empathic prosocial behavior, and further, that this depends on the context in which the discussions take place.

### Socializing prosocial behavior by discussing emotions and mental states

Toddlers' prosocial behavior toward an adult in distress, in this case offering something to alleviate the distress, was associated with parents' emotion and mental state discourse with their children both while reading books together and while playing with toys together. The specific pathways through which discourse about emotions and mental states may operate to encourage prosocial behavior remain unknown. Emotion understanding, given its robust associations with EMST, is one logical pathway (de Rosnay and Hughes, 2006; Thompson, 2006). Greater emotion understanding promoted by frequent parental EMST may help children infer the other's need and generate an appropriate prosocial response in a complex, affectively charged situation (Denham et al., 2007; Ensor et al., 2011; Brownell et al., 2013). Emotion and mental state discourse may also operate on children's prosocial motivations by facilitating empathy inductions, leading to internalized moral dispositions that underlie prosocial behavior toward those in distress (Hoffman, 2000). A third possibility is that parents' EMST encourages toddlers to attend to the emotions and mental states of others, which in turn provides more exposure and, consequently, more opportunity to construct meaning. Further work is necessary to determine which pathway is the most likely; but regardless of the pathway or pathways through which it operates, discourse about emotions and mental states appears to play an important role in the early socialization of prosocial behavior.

However, this role varies by context. We largely replicated the findings from Brownell et al. (2013) that it is parents' elicitation of children's own EMST in the context of book reading (e.g., “how is he feeling?” “is he sad?”), rather than parents' labeling and explaining, that relates to toddlers' empathic prosocial behavior. Replication of these findings with different parent-child dyads, different adults in need of help, and different picture books strengthens the conclusion that discourse about emotions and mental states is likely to influence early prosocial development most effectively when parents ask children themselves to think about and explain others' emotions. Findings from the joint-play context of the current study expand and refine this conclusion, adding to our understanding of the role of EMST in relation to developing prosociality. Specifically, we found that parents' labeling and explaining of emotions and mental states in that context (e.g., “he wants to go in the barn,” “you must like that toy”), rather than elicitation of children's talk, was related to empathic prosocial behavior. Thus, although the relation between parent-infant discourse about emotions and mental states and early prosocial behavior is a general one, the particular features of the discourse that appear to matter are context-specific.

There are several potential explanations for these context differences. As compared to joint play with toys, reading a picture book provides the child with specific emotion referents as well as overt cues to help her identify and piece together characters' internal states, often including exaggerated emotional facial expressions and a plot or narrative that helps place the character and the emotion-eliciting events in meaningful context. These cues can be made salient to the child by the parent, especially by asking the child to attend to and reflect on them, and may help her understand the character's mental state, the factors that led to that state, any consequences of that state, and how the state can be alleviated. When asked questions that require the child to recruit her rudimentary social understanding, she may need and utilize all of these cues to make sense of the question. Without such cues, questions about internal states may be meaningless or overwhelming; with them, children may be able to find answers, and construct social knowledge in the process.

Additionally, the referent of parents' EMST may differ between contexts, with parents more often talking about the emotions and mental states *of others* (i.e., characters) during book reading and *of the child* during joint play (e.g., “Oh, you want to play with that”). Although we did not measure the parents' referents, previous work has found precisely this difference, with parents using more EMST about their child during joint play and about others while reading a book (Beeghly et al., 1986). At this age, a toddler's immature ability to reflect on herself and regulate her emotions may preclude her from being able to respond to questions about what she is feeling or thinking while in the midst of playing (Thompson and Goodvin, 2007); discussing the emotions of others pictured on the pages of a book, on the other hand, may be an easier task as it does not depend on reflective self-awareness and emotion regulation to the same degree. Consequently, parents' commentary about emotions and mental states may be more effective in scaffolding young children's emerging social understanding when references are made to the child's own mental states, while parents' attempts to elicit the child's talk would be more effective when references are made to the mental states of others. An alternative view is that children's understanding of the mental states of others derives from understanding of their own mental states, suggesting that conversation about one's own mental states may be easier for toddlers (Taumoepeau and Ruffman, 2006, 2008), hence that toddlers are more equipped to answer questions about their own mental states than about others'. Our data is more consistent with the former interpretation than the latter (see Carpendale and Lewis, 2004, for a discussion of potential problems with the latter interpretation), but as we did not measure the referents of parent EMST talk we are unable to address this question directly. Future work is needed to assess which of these interpretations more accurately explains associations between parent talk and early prosocial competence.

## Limitations and conclusions

Findings from this study are subject to several limitations. The correlational design precludes firm causal inferences about the effects of EMST on prosocial development, and the cross-sectional design precludes inferences about the long-term and cumulative effects of greater relative EMST use. Although our findings are consistent with conceptual frameworks positing the causal influence of parental socialization, experimental and/or longitudinal designs are required to make such inferences with confidence. Additionally, the current study included only parents' talk. To fully understand and appreciate the contextual differences in these joint activities, the child's input must be considered. It will also be important in future work to explore differences between mothers and fathers in EMST use and corresponding relations with prosocial behavior. The generalizability of the findings is also limited. We hoped to capture snapshots of parent-child interactions as they would unfold in the home during ecologically valid dyadic activities like joint play and book reading, but this may have been constrained by the lab atmosphere. Naturalistic work conducted in the home would provide converging evidence. Finally, it is unknown whether the patterns we have identified generalize across cultures or across families differing in education, family income, or other socioeconomic indicators.

The findings of the current study contribute to the growing literature examining discourse about emotions and mental states as a mechanism through which parents are likely to socialize prosocial behavior. Parent- child interaction dominates children's first few years, providing many opportunities for discussing emotions and mental states beginning very early in life (Dunn, 1988; Meins et al., 2001). The structure and tone of these interactions vary widely, but each conversation offers the child a context in which to explore and begin to understand the complexities of subjective states. In the current study we have shown that discourse about emotions and mental states in two different contexts is related to children's prosocial behavior. Although parents use different types of EMST depending on the context, developmentally appropriate usage, with sufficient scaffolding, appears to be an effective way for parents to promote other-oriented prosocial responding in their very young children.

### Conflict of interest statement

The authors declare that the research was conducted in the absence of any commercial or financial relationships that could be construed as a potential conflict of interest.
