# Supplementary material for: Here, there and everywhere: emotion and mental state talk in different social contexts predicts empathic helping in toddlers
Source: Front Psychol. 2014 Apr 29;5:361. doi: 10.3389/fpsyg.2014.00361 (PMC4010777; doi:10.3389/fpsyg.2014.00361)
Supplement: Supplementary file 1 [file Presentation1.PDF]

Appendix

Coding Criteria for Parents' Emotion and Mental State Talk

Category	Examples	
	**Production**	**Elicitation**
**Simple Affect**<br>Nouns, verbs, adjectives, or adverbs naming emotional feelings or behaviors, or states of preference, desire, or intention without expansion or emotion imitation	Production (SAP)<br>• The boy is happy.<br>• He loves his ice cream.<br>• You like this toy.	Elicitation (SAE)<br>• Is he happy or sad?<br>• How is he feeling?<br>• Are you happy?
**Desire**<br>References to wanting, needing something concrete	Production (DP)<br>• He wants his ice cream.<br>• You want that toy.	Elicitation (DE)<br>• Does he need a hug?<br>• What do you want to play with?
**Elaboration/Explanation**<br>Phrases or statements that explain or clarify the reason or possible cause for a particular mental state, or that provide background or context to help the child understand it, or that elaborate or explain how one infers or knows that a given mental state is being experienced	Production (EP)<br>• The monkey is sad because he doesn't have a hug.<br>• The girl is scared because it is dark.<br>• You're excited because we get to play with these toys.	Elicitation (EE)<br>• Why is the monkey sad?<br>• How do you know the girl is angry?<br>• Why are you upset?
**Mental state**<br>References to the past, or to thinking, knowing, wondering, remembering, pretending	Production (MSP)<br>• I think they're lizards.<br>• You know this color.	Elicitation (MSE)<br>• What do you think they're doing?<br>• Do you remember reading about elephants in our story?
**Other Internal State**<br>References to other internal states that are not affect- or mental state-related (e.g., physiological states)	Production (OISP)<br>• He is hungry.<br>• You seem tired.	Elicitation (OISE)<br>• Did she get tired?<br>• Are you hungry?
**Empathy Statements**<br>Statements or emotion-related sounds that promote empathy with a character's emotion	Production (EMPP)<br>• Aww.<br>• Poor monkey.	Elicitation (EMPE)<br>* No instances of EMPE occurred
